# Novel treatment of traumatic CSF rhinnorhea using titanium mesh and onlay graft

**DOI:** 10.4103/1817-1745.66678

**Published:** 2010

**Authors:** Pankaj Ailawadhi, Deepak Agrawal, A. K. Mahapatra

**Affiliations:** Department of Neurosurgery, All India Institute of Medical Sciences, New Delhi - 110 029, India

**Keywords:** Cranial defect, CSF rhinnorhea, pericranial graft, repair, titanium mesh, trauma

## Abstract

Post-traumatic rhinnorhea due to large frontobasal fractures remains a difficult entity to treat. The authors report the case of a 9-year-old boy who had persistent CSF rhinnorhea due to extensive frontobasal fractures and who was managed with transcranial extradural surgery with titanium mesh placement and only pedicled pericranial flap.

## Introduction

The appropriate timing as well as type of surgical intervention in patients with persistent post-traumatic CSF leaks remains unclear. Many surgical approaches – both intracranial and extracranial – may be successful, depending on patient factors and the anatomy of the fistula. The goal of neurosurgical management of CSF rhinorrhea is to prevent external deformity of the skull, seal the CSF leak, and to avoid chronic sinusitis. The best method of closing frontobasal defects is still a matter of debate, especially if there is a need for reconstructive surgery.[1–5] We report a case of post traumatic CSF rhinorrhea resulting from extensive dural tears and anterior cranial fossa floor fractures and its subsequent repair using titanium mesh and pedicled pericranial flap.

## Case Report

**Figure 1:**
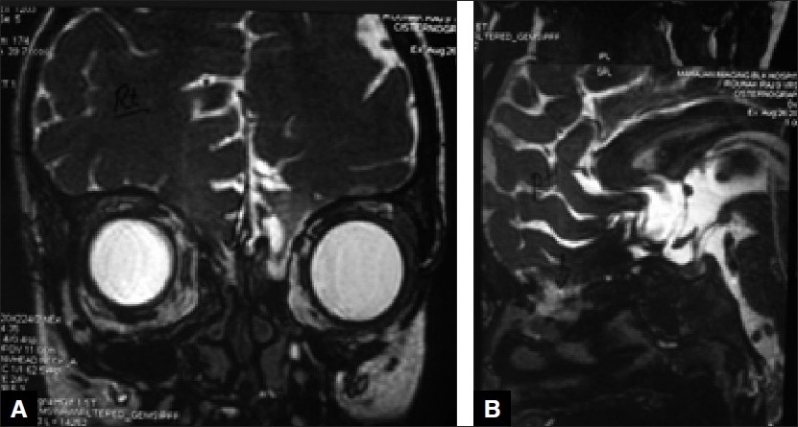
MRI cisternography coronal (A) and sagittal (B) views showing the bilateral cribriform plate defects

**Figure 2:**
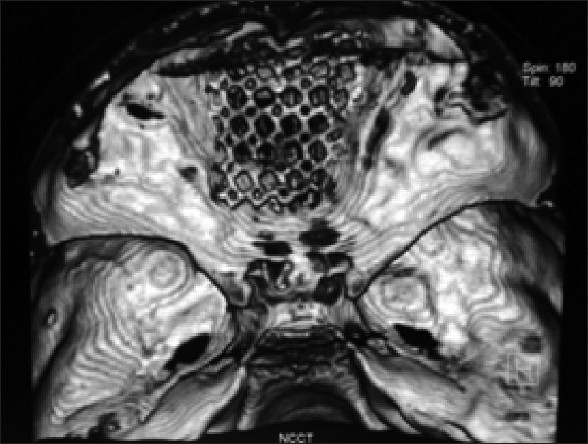
Postoperative CT head with 3D reconstruction showing reconstruction of anterior cranial floor with the titanium mesh

A 9-year-old boy presented to us with complaints of watery nasal discharge from right nostril after seven months following fall from 20 feet. He had suffered four episodes of high grade fever in the intervening period. On presentation, he was fully conscious without any neurological deficits. An MRI cisternography was done which showed extensive bilateral cribriform plate defects with a doubtful defect from the sellar floor into the sphenoid sinus [Figure 1]. The patient was taken for surgery and a bicoronal flap was raised. A bipedicled pericranial flap was raised for use as an onlay graft. Multiple dural defects were noted in bilateral basifrontal region with large bilateral cribriform plate defects through which the frontal lobe was herniating. The herniating brain tissue was released from these outpouchings with sharp dissection. Dural defects were repaired primarily. In view of large bilateral bony defects, it was decided to reinforce the anterior cranial fossa floor with titanium mesh which was contoured and placed over the anterior skull base. The mesh was fixed to the floor [Figure 2] with the help of 4 mm screws. An onlay graft of the pedicled pericranium flap was interposed between the mesh and the dura using fibrin glue.

A lumbar drain was placed prophylactically and CSF drainage was continued till third post operative day.

The remainder of the hospital course was uneventful and the patient could be discharged on the sixth post operative day and remains asymptomatic at last follow up.

## Discussion

The use of titanium mesh in skull base surgery has previously been reported in craniofacial and cranial vault procedures.[1–4] Its use in skull base applications may prove useful in certain situations. There are a variety of options for techniques to repair the defect of the anterior skull base, but the principle concept is to have a ‘water-tight’ closure.

Badie[5] evaluated the usefulness of titanium mesh along with two layered pericranium flap in reconstruction of anterior cranial fossa floor in 13 patients and found that none of the patients had complications like infections or meningocele formation at a mean follow-up of 22 months. The reconstruction technique involved placement of titanium mesh between two layers of continuous vascularized pericranium. We, however, placed the titanium mesh inferior to the two layers of continuous vascularized pericranium to prevent injury to the pericranium by the mesh.

## Conclusion

Our case shows that the use of titanium mesh is a safe and feasible method for reconstructing large anterior skull base.
